# Efficacy of Feeding Grape By-Products on Performance, Nutrient Digestibility, Gut Morphology, Gut Microbial Community, Oxidative Stress and Immune Response in Fast-Growing Broilers

**DOI:** 10.3390/ani15131943

**Published:** 2025-07-01

**Authors:** Robert Ringseis, Klaus Eder, Denise K. Gessner

**Affiliations:** 1 Institute of Animal Nutrition and Nutrition Physiology, Justus Liebig University Giessen, Heinrich-Buff-Ring 26-32, 35392 Giessen, Germany; klaus.eder@ernaehrung.uni-giessen.de (K.E.); denise.gessner@ernaehrung.uni-giessen.de (D.K.G.); 2 Center for Sustainable Food Systems, Justus Liebig University Giessen, Senkenbergstraße 3, 35390 Giessen, Germany

**Keywords:** grape by-products, polyphenols, performance, digestibility, inflammation, oxidative stress, fast-growing broilers, gut microbiota, gut integrity

## Abstract

By-products from winemaking, such as grape pomace, grape seeds, grape skins, or extracts made from them, represent a cost-effective and sustainable bioresource. These by-products are a source of polyphenolic compounds, plant fibers and—in the case of seeds—essential fatty acids with various health-promoting effects for livestock. Numerous studies involving fast-growing broiler breeds—which often suffer from metabolic inflammation and oxidative stress due to disproportionate breast muscle growth leading to issues like cardiorespiratory insufficiency—indicate that supplementing feed with grape by-products improves performance, particularly weight gain and feed efficiency. This literature review demonstrates that the performance-enhancing effects of grape by-products in fast-growing broiler breeds can be attributed to various mechanisms such as improved nutrient digestibility, a positive influence on intestinal morphology and integrity, the favorable modulation of the microbial community in the gut, the inhibition of oxidative stress or the enhancement of the antioxidant defense system, and the stimulation of the immune response.

## Simple Summary

The utilization of agricultural by-products obtained from industrial food processing, such as wine production, as a feed source is a reasonable strategy to increase the sustainability of livestock production. This strategy reduces the additional resources required for feed production (e.g., land, water) while minimizing the environmental impact of livestock farming. By-products from wine production are of particular interest as sustainable feed sources because they not only enhance the sustainability of livestock production through reducing the utilization of other feed sources but also have a positive impact on animal health. This literature review demonstrates that supplementing the diet of fast-growing broilers with grape by-products improves their growth performance through different mechanisms, including the improvement of nutrient digestibility, a positive influence on gut health, the favorable modulation of the microbial community in the gut, the inhibition of oxidative stress and the stimulation of the immune response.

## 1. Introduction

Using agricultural by-products from industrial food processing—such as wine and juice production, beer brewing, grain milling, and oil extraction—as a feed source is an effective strategy to enhance the sustainability of livestock production. This approach reduces the demand for additional resources like land and water for feed cultivation while minimizing the environmental impact of livestock farming [1]. Increasing the sustainability, environmental compatibility and resource-efficiency of animal production is an urgent need; this is because the global demand for food and feed production is strongly increasing on the one hand, but natural resources are becoming increasingly limited due to land degradation, water shortage, climate change and other reasons on the other hand [2,3]. Large amounts of agricultural by-products accumulate during wine production in various wine-producing regions worldwide. In the EU, Italy, France, and Spain are the leading wine-producing countries, collectively contributing to approximately 50% of global wine production [4]. Grape pomace—the solid residue obtained after juice extraction from grapes and consisting of skins, seeds and stems—is the predominant by-product of winemaking, making up 20–30% of the grape mass [5]. Since the disposal of grape pomace in open fields near wineries can lead to significant environmental issues, such as soil and water contamination [6], incorporating grape pomace and other winemaking by-products (e.g., stems, lees) into a circular economy is essential. In this regard, utilizing grape by-products as a feed source presents a viable strategy for managing agricultural waste from winemaking and enhancing the sustainability of livestock production by reducing reliance on less sustainable feed sources. Currently, grape pomace is the most widely used grape by-product for livestock feeding. It is utilized either in fresh form in winemaking areas in Germany and other EU countries as a feed component in total and partial mixed rations for ruminants or in dried or extracted form as a phytogenic feed component in compound feed for monogastric farm animals.

Grapes contain various beneficial bioactive constituents, wherefore the use of grape by-products as a feed source also has a positive impact on animal health [7,8,9]. Grapes—especially the skins and stems—contain different indigestible carbohydrates (fibers) [7], which are known to affect digesta viscosity and the passage rate, as well as the structure of the gut microbial community, and, thereby, gut integrity and gut function. In addition, grapes are a rich source of a wide variety of secondary plant compounds, particularly phenolic compounds (e.g., anthocyanins, flavanols, flavan-3-ols, procyanidins, phenolic acids, resveratrol) [5,10]. Since only 30–40% of the phenolic compounds from grapes transfer into the wine, while the greater part remains in the grape pomace [11,12], grape by-products are also a phenolic compound-rich feed source. Since phenolic compounds are unevenly distributed within different parts of the grape, the concentrations and the type of phenolic compounds vary among different grape by-products. While the seeds have the highest concentrations of total phenolic compounds and total flavan-3-ols, the concentrations of total flavonols, total phenolic acids and total anthocyanins are significantly higher in the skins than in the seeds [11]. Consequently, the concentrations of these phenolic fractions in grape pomace lie between those found in grape seeds and grape skins [11].

Amongst the beneficial biological activities associated with the consumption of grapes and grape by-products, the inhibition of inflammatory processes and oxidative stress are particularly well-documented. In this context, it is worth mentioning that both inflammation and oxidative stress are common in high-producing farm animals, and that the supplementation of polyphenolic compounds from plant materials is an effective strategy for reducing inflammation and oxidative stress in livestock, as extensively reviewed by Gessner et al. [13]. Inflammatory processes not only affect animal health but also economic efficiency and environmental sustainability, because inflammation renders animal production less efficient due to impaired feed conversion [14,15,16], reduced feed intake [17,18,19], and damage to the intestinal barrier [20,21,22].

Amongst the high-producing farm animals known to be particularly affected by inflammatory processes are modern, fast-growing (“meat-type”) broilers [23,24], which have been selected for their improved feed conversion, accelerated growth, carcass weight, and breast muscle yield [25]. In modern meat-type broilers, up to 30% of their body weight (BW) is accounted for by breast muscles, which—originally consisting of red, “oxidative” fibers adapted for flying—have been modified to contain predominantly white, “glycolytic” fibers to facilitate hypertrophic growth [26]. This disproportional growth of breast muscle in meat-type broilers is accompanied by impaired cardiopulmonary function, acidosis, mitochondrial dysfunction and oxidative stress [27,28], as well as an inflammatory process that promotes the fibrotic replacement of muscle fibers with connective tissue [29,30]. These structural changes manifest as different necrotic/fibrotic myopathies (e.g., Wooden Breast, Spaghetti Meat), which have been identified in modern meat-type broilers with high prevalence and are associated with an impaired breast meat quality [31]. Interestingly, myopathies, such as Wooden Breast, were also shown to be correlated with a higher number of adipocytes and more ectopic fat depots [32]—conditions which are well-known to be associated with metabolic inflammation and oxidative stress. Overall, these observations suggest that fast-growing broilers display an unfavorable metabolic phenotype that could benefit from the antiinflammatory and antioxidant properties of grape by-products.

In light of the above, the present review aims to evaluate the efficacy of grape products as feed additive for modern, meat-type broiler breeds, such as Ross, Cobb, Arbor Acres and Hubbard, by systematically analyzing studies reporting the effects of grape by-products on performance, nutrient digestibility, gut morphology and integrity, the gut microbial community, oxidative stress and antioxidant status, and the immune response in fast-growing broilers.

## 2. Methods

The present review is based on a literature search using the PubMed database (NCBI, Bethesda, MD, USA) that was conducted in March 2025. Potentially suitable original research papers were identified using “grape”, “grape by-products”, “grape products”, “broilers”, “performance”, “digestibility”, “intestinal morphology”, “gut microbiota”, “antioxidant status”, “oxidative stress”, “immune response” and “inflammatory mediators” as search terms. Out of the 65 studies identified, a total of 44 studies, which were performed with meat-type broiler breeds, such as Ross, Cobb, Arbor Acres or Hubbard, were found suitable to address the research questions of the review.

## 3. Effects of Grape By-Products on Fast-Growing Broilers

### 3.1. Effect of Grape By-Products on Growth Performance

A large number of studies have examined the effect of grape by-products on broiler performance. The most important study characteristics (broiler breed, sex, product dose, polyphenol concentration, duration) and the outcomes are summarized in Table 1.

The studies showing an improvement in performance slightly outweigh those reporting either neutral or negative effects on performance. Amongst the studies showing improvements, it was demonstrated that feeding a diet supplemented with a grape seed extract (0.1 g/kg diet) to Ross 308 broilers from 1 to 42 days of age increases the daily feed intake and BW gain and reduces the feed:gain ratio [33]. Likewise, BW gain and feed intake were found to be increased by feeding Ross 308 broilers diets supplemented with 20 and 30 g of grape seed powder per kg diet from 22 to 42 days of age [35]. Also, feeding Arbor Acres broilers with a diet comprising a herbal extract blend containing grape seeds (1.5 g/kg diet) increased BW gain and improved the feed efficiency compared to broilers fed no herbal extract blend [36]. However, since the herbal blend also contained other plant components (e.g., hop), the contribution of grape seeds to the performance-improving effect is uncertain. Daily BW gain was also increased in Ross 308 broilers when feeding them a diet supplemented with a grape pomace extract (2 g/kg diet) from days 1 to 44 of age, whereas feed intake and the feed:gain ratio were not affected by the dietary treatment [38]. Feeding female Ross 308 broilers a diet supplemented with either 5 g of unfermented or 5 g of fermented grape seeds per kg diet for 42 days increased BW gain but did not influence feed intake and the feed:gain ratio compared to those fed the unsupplemented diet [43]. In a second study by Gungor et al. [44], feeding Ross 308 broilers a diet supplemented with 15 g of fermented grape pomace per kg diet but not with 15 g of unfermented grape pomace per kg diet for 42 days increased the final BW. Feed intake and the feed:gain ratio in this study were not affected in comparison to those fed the unsupplemented diet. The fact that only the fermented grape pomace showed a growth-promoting effect may be attributed to the formation of antimicrobial compounds during fermentation with *Aspergillus niger*, as well as the resulting beneficial shift in the composition of the gut microbiota—fewer pathogenic bacteria and more beneficial ones [44]. The dietary supplementation of Arbor Acres Plus broilers with a grape seed extract at two doses (0.2 and 0.4 g/kg diet) for 3 weeks increased BW gain and decreased feed intake and the feed:gain ratio [45]. In another study, the inclusion of 10 and 20 g/kg diet of grape seeds into a broiler diet dose-dependently increased BW gain and decreased the feed:gain ratio of Cobb 500 broilers after 42 days, whereas the performance of broilers was reduced at an inclusion level of 40 g grape seeds per kg diet [50]. Probably, at the highest inclusion level for grape seeds, the high amount of total phenolic compounds (2.23 g/kg diet) exerted antinutritional effects. Yang et al. [52] fed a diet supplemented with either 7.5, 15 or 30 mg of grape proanthocyanidins per kg diet to Cobb 500 broilers for 42 days and observed no effect on BW gain but found an improved feed conversion (reduced feed:gain ratio) due to a decreased feed intake. The authors did not provide an explanation for the decrease in feed intake due to supplementation with grape proanthocyanidins, but it may be related to the impairment of feed taste due to the dietary inclusion of proanthocyanidins. Beneficial effects on broiler performance were not only found under normal conditions but also under different challenge conditions. For instance, feeding an aflatoxin B1-contaminated diet supplemented with either 0.25 or 0.5 g grape seed extract per kg diet to Cobb broilers for 4 weeks abrogated the reduction in feed intake and BW gain and the increase in the feed:gain ratio induced by feeding them the unsupplemented aflatoxin B1-contaminated diet [49]. In addition, in broilers artificially infected with *Eimeria tenella*, feeding them diets with different concentrations of a grape seed extract (5, 10, 20, 40 and 80 mg/kg diet) increased BW gain in comparison to infected broilers fed the unsupplemented diet [57]. This indicates that feeding broilers with grape by-products is a strategy able to mitigate the negative impact of different challenging conditions on the growth performance of broilers. In two further studies, in which treatments combining a grape product with another feed additive were investigated, improvements in broiler performance were observed. In one of these studies, broilers fed a red grape pomace-containing diet (30 g/kg diet) and administered Aloe vera via drinking water (10, 20 and 30 g/L) were found to have a higher BW gain and a lower feed:gain ratio than broilers fed no red grape pomace and administered no Aloe vera [34]. In another study, the combined feeding of Hubbard broilers infected with *E. tenella* with organic zinc (50 mg/kg diet) and grape seed powder at 2.5 and 5 g/kg diet increased feed intake and BW gain and decreased the feed:gain ratio during the whole feeding period (4 weeks) compared to *E. tenella*-infected broilers fed no feed additives [42]. Owing to the combined treatment strategy, the contribution of the grape by-products to the effects observed cannot be evaluated.

In contrast to the above-mentioned studies, no effect on feed intake, BW gain and the feed:gain ratio during the whole period (d 1–40) was found in Ross 308 broilers fed a diet supplemented with 2 g of olive leaf and grape-based by-product per kg diet compared to broilers fed the unsupplemented diet [37]. It cannot be ruled out that constituents of the olive leaf, such as crude fiber, may have counteracted the potentially positive effect of the grape-based by-product on performance, as crude fiber exerts antinutritional effects at higher concentrations. Also, feeding a corn–soybean basal diet supplemented with either 5, 15, and 30 g of grape pomace per kg diet did not affect BW gain, feed consumption and the feed:gain ratio in male Cobb broilers in comparison to those fed the unsupplemented basal diet [59]. In addition, in a study in which Ross 308 broilers were fed either a basal diet or a basal diet supplemented with grape pomace (25 g/kg), wine lees extract (2 g/kg) or pure stem extract (0.1 g/kg) for 42 days, no effects on the final BW, feed intake and feed:gain ratio were observed [40]. Moreover, no effect on BW gain, feed intake and the feed:gain ratio was observed in response to feeding diets supplemented with either 5, 7.5 or 10 g of grape pomace per kg diet to male Ross 308 broilers for 4 weeks in comparison to those fed the unsupplemented diet [48]. Furthermore, no effect on growth performance was seen in Ross 308 broilers fed diets supplemented with either 0.125, 0.25, 0.5, 1 or 2 g of grape seed extract per kg diet for 42 days [51]. Likewise, no effect on feed intake, the final BW and feed:gain ratio was found in Cobb 500 broilers administered a plant extract containing red grape pomace via drinking water (1 g/L) for 6 weeks [53]. A study by Cross et al. [55] also revealed that dietary supplementation with a grape seed extract (1 g/kg diet) had no effect on female Ross 308 broilers at days 22–42 of age regarding their BW gain, feed intake and feed:gain ratio. Also, Brenes et al. [58] observed that the dietary inclusion of grape pomace concentrate at 15, 30, and 60 g/kg had no effect on male Cobb broilers at days 21–42 of age regarding their BW gain, feed intake and feed:gain ratio. In neither of the above-mentioned studies [40,48,51,53,55,58,59] was the influence on gut morphology or gut microbiome examined. Therefore, it cannot be assessed whether the lack of improvement in growth performance is due to the absence of enhancements in gut morphology or the gut microbiome. However, in one study by Erinle et al. [39], in which BW gain and the feed:gain ratio were also not influenced by feeding a diet with grape pomace (25 g/kg diet) to Cobb 500 broilers from days 1 to 42 of age, gut morphology and gut microbiota composition showed improvements but these positive effects did obviously not improve growth performance.

Finally, some broiler studies reported the impairment of performance in response to feeding grape by-products. One of these is the study by Romero et al. [41], in which a reduced BW gain and an increased feed:gain ratio was found in Cobb broilers fed a diet supplemented with grape skin meal (110 g/kg diet). In contrast, feeding broilers a diet supplemented with either grape seed meal (30 g/kg diet) or a mixture of grape seed meal (24.4 g/kg diet) and grape skin meal (13.1 g/kg diet) had no effect on growth performance in this study [41]. Considering that the three diets contained similar amounts of total extractable grape polyphenols but that the concentration of non-extractable polyphenols was higher in the grape skin diet than in the other two diets, the growth-depressive effect of the grape skin meal is likely explained by the higher amounts of non-extractable polyphenols (polymeric phenolic compounds) and their negative effect on protein digestibility. In agreement with this, the broilers fed the grape skin meal diet had a decreased ileal protein digestibility [41]. In another study, the inclusion of a grape seed extract at 0.025, 0.25 and 2.5 g/kg diet for 3 weeks did not affect the performance of Cobb broilers, whereas the inclusion of 5 g of grape seed extract per kg diet reduced BW gain and impaired feed conversion (increased feed:gain ratio) [54]. This clearly shows that polyphenolic compounds from grape by-products exert a growth-depressing effect above a certain threshold concentration, which was between >0.75 and 1.5 g gallic acid equivalents/kg diet in the present study. However, the results of Brenes et al. [60], in which a concentration of 1.5 g gallic acid equivalents/kg diet resulting from the inclusion of a different commercial grape seed extract did not affect growth performance [60], suggest that the effect on growth performance is not only dependent on the concentration of total polyphenols in the diet but also on the dietary polyphenolic profile. In addition, a negative effect on growth performance was reported in a study by Nardoia et al. [46], in which feeding a diet supplemented with 60 g of either fermented or unfermented grape skins for 3 weeks decreased BW gain and increased the feed:gain ratio of Cobb broilers in comparison to those fed the unsupplemented diet. Considering that the dietary concentration of gallic acid equivalents was 2.27 and 3.13 g/kg diet in the fermented and the diet, respectively, it can be assumed that this dose of polyphenols caused a growth-depressive effect in the broilers. Moreover, a reduction in BW gain and an unaltered feed:gain ratio was seen in Cobb 500 broilers fed a diet supplemented with 100 g of grape pomace per kg diet for 6 weeks [47], probably due to the high dose of polyphenolic compounds fed through the grape pomace. However, when the grape pomace was pretreated with polyethylene glycol to bind tannins, the diet supplemented with 100 g/kg diet of polyethylene glycol-pretreated grape pomace did not impair growth performance when compared to the unsupplemented diet. Negative effects on BW gain were also observed when feeding a basal diet supplemented with a grape seed extract (7.2 g/kg diet) to male Cobb broilers for 3 weeks [56]. However, in the same study, a basal diet supplemented with grape pomace concentrate (60 g/kg diet) did not impair BW gain and even decreased the feed:gain ratio of broilers compared to broilers fed the unsupplemented diet. Since the concentrations of extractable polyphenols were similar between both diets, the growth depression could be attributed to the specific polyphenolic profile of the grape seed extract.

Taken together, it can be summarized that studies showing improvements and studies showing either neutral or negative effects of grape-products on broiler performance are relatively balanced. While low doses of grape by-products improve growth performance through improving gut morphology, the composition of the gut microbiota and gut function, as well as attenuating inflammatory processes and oxidative stress, high doses of grape by-products can impair broiler performance due to high amounts of antinutritional compounds, such as polyphenols and indigestible carbohydrates.

### 3.2. Effect of Grape By-Products on Nutrient Digestibility

It has long been known that polyphenols, particularly condensed tannins, have the ability to aggregate and precipitate proteins and impair macronutrient utilization by forming tannin–protein complexes with both dietary and endogenous proteins. This contributes to the observation that polyphenol-rich feedstuff, such as faba beans and sorghum, reduce protein and amino acid digestibility in broilers and other monogastric species [61,62]. Owing to this, polyphenols have been recognized primarily as antinutritional factors negatively affecting animal performance by impairing nutrient availability. Despite the great relevance of nutrient digestibility for broiler performance, surprisingly few studies dealing with grape by-products as feed additives have investigated the effect on nutrient digestibility in fast-growing broilers. An overview of these studies is given in Table 2.

In line with the above-mentioned mechanisms of polyphenols, an impairment of the apparent ileal digestibility of crude protein and several amino acids was found in male Cobb broilers fed a commercial aqueous grape seed extract at 5 g/kg diet (corresponding to 0.15 g gallic acid equivalents per kg diet) for 3 weeks [54]. No effect was seen at lower concentrations of the grape seed extract (0.25 and 2.5 g/kg diet) in this study, indicating that the antinutritive effect of polyphenols occurs only at higher doses. In another study from the same group, it was shown that grape pomace at a high concentration of 100 g/kg diet (corresponding to 0.2 g gallic acid equivalents per kg diet) decreases ileal protein digestibility in male Cobb broilers, whereas no adverse effect was observed at a lower dose [63]. In the latter study, it was also demonstrated that the addition of exogenous tannase (tannin acyl hydrolase) decreases ileal protein digestibility, at least when added to the diet with a high concentration of grape pomace. This observation has been explained by the fact that tannase hydrolyzes polymeric phenolic compounds present in grape pomace, as shown in vitro [64], thereby releasing monomeric and also dimeric compounds (procyanidins B) in the ileal digesta [63]. This accumulation of phenolic compounds in the ileal digesta is supposed to increase the interactions among polyphenols and proteins in the intestine, thereby reducing protein digestibility [63]. In line with studies by Chamorro et al. [54,63], a reduction in ileal protein digestibility was demonstrated in male Cobb broilers fed two different doses of unfermented grape skins (30 and 60 g/kg diet), whereby this effect was more pronounced in broilers fed the higher dose [46]. Interestingly, ileal protein digestibility was not impaired in this study when fermented grape skins were fed to broilers at the same dose as the unfermented ones [46]. During fermentation, a number of compounds, such as polysaccharides, mannoproteins and polyphenols, are released from the solid grape residues [65]. In line with this, the content of phenolic extractable compounds was found to be lower in the fermented than in the unfermented grape skins [46]. Thus, the observation that the unfermented grape skins negatively affected the protein digestibility of the broilers is probably explained by the higher concentration of total extractable polyphenols in the unfermented than in the fermented grape skins and the interaction between these polyphenols and dietary protein. In a further study on male Cobb broilers, feeding them diets with grape skins (110 g/kg diet) obtained from red grape pomace was reported to decrease not only the ileal digestibility of protein but also that of extractable polyphenols [41]. Interestingly, feeding broilers with a grape seed-supplemented diet with the same concentration of grape polyphenols as the diet with grape skins did not reduce ileal protein digestibility. This difference between grape skins and grape seeds has been attributed to the fact that the diet with grape skins contained a much higher concentration of non-extractable polyphenols, such as proanthocyanidins, phenolic acids and hydrolysable tannins [41]. Such polyphenols are supposed to exert a stronger inhibitory effect on protein digestion due to their higher polarity compared with other polyphenols [66]. This enables them to form more complexes with proteins due to the interaction of their hydroxyl groups with the carbonyl groups of proteins [41]. Thus, these findings clearly show that the effect of polyphenols on nutrient digestibility is not only dependent on the dose but also on the type of polyphenols, which largely differs between distinct grape products.

In contrast to the above-described studies, the apparent total tract digestibility of crude protein, crude fat and energy was not decreased in Ross 308 broilers fed a basal diet supplemented with either 5, 7.5 or 10 g grape pomace per kg diet compared to broilers fed the unsupplemented basal diet for 28 days [48]. Based on the analyzed content of total polyphenols in the grape pomace reported in this study [48], the polyphenol concentration in the diets with the highest inclusion of grape pomace was markedly lower than that in the study by Romero et al. [41]. Thus, it is likely that the lack of an inhibitory effect on protein digestibility when feeding broilers with grape pomace compared to the study by Romero et al. [41] is due to the difference in the dietary polyphenol concentration. Apart from the study by Aditya et al. [48], no effect on the apparent ileal digestibility of protein was reported in male Cobb broilers fed diets supplemented with either 15, 30 or 60 g grape pomace concentrate per kg diet compared to the unsupplemented control diet for 3 weeks [58]. However, the apparent ileal digestibility of fat was found to be reduced by the grape pomace concentrate at 30 and 60 g/kg diet compared to the unsupplemented control diet. Brenes et al. [58] also supposed that the lack of effect on protein digestibility is attributed to the low content of polyphenols in the experimental diets. The inhibitory effect of the grape pomace on fat digestibility was proposed by the authors to be associated with an increase in lipid excretion, as observed earlier in rats [67], because condensed tannins have been shown to bind biliary salts and cholesterol, with a concomitant reduction in their absorption and an increase in fecal excretion [68]. Like in the study by Brenes et al. [58], no effect of feeding broilers a condensed tannin extract from grape seeds at 1 g/kg diet on ileal protein digestibility compared to feeding broilers an unsupplemented control diet was observed in female Ross 308 broilers [55]. Moreover, a study with male Cobb broilers demonstrated no reduction in the apparent ileal digestibility of crude protein and essential and non-essential amino acids in response to feeding graded concentrations of red grape pomace (5, 15 and 30 g/kg diet) for 3 weeks [59].

Beyond the effects on macronutrient digestion, the effects of feeding a commercial aqueous grape extract at two doses (2.5 and 5 g/kg diet) on the apparent ileal and total tract digestibility of different grape polyphenolic compounds was investigated in male Cobb broilers [69]. Based on this study, the ileal digestibility of monomeric flavan-3-ol compounds (catechin, epicatechin) was very high (84–87%) in broilers fed the two diets with the grape extract. The ileal digestibility of procyanidin dimers B1 and B2, and the galloylated compound epicatechin-*O*-gallate, was markedly lower, being in the range of 50–69%. In agreement with this, the results of another study from the same group showed that the monomers and dimers of grape procyanidins are highly digestible along the intestinal tract of broilers, whereas epicatechin-*O*-gallate is less digestible [63]. Likewise, a low digestibility of polymeric grape phenolic compounds was found in Cobb broilers fed grape-pomace-concentrate-supplemented diets [58]. Although the reported digestibility data indicate that monomeric polyphenolic compounds, unlike galloylated forms, are systemically available, thereby becoming bioefficient in some tissues, it has to kept in mind that a high digestibility does not necessarily translate to a high systemic bioavailability. Digestibility only accounts for the disappearance of the ingested grape polyphenolic compounds from the intestine, which could result from degradation, biotransformation, or absorption. In fact, the observation that a wide range of low-molecular-weight phenolic compounds, such as benzoic acids, propionic acids, cinnamic acids, as well as valerolactones derivatives, are found in the excreta of broilers fed the grape extract [69] suggests that an important proportion of the ingested grape catechins disappear and/or are chemically modified throughout the intestinal tract of chickens.

Overall, it can be summarized that high doses of dietary grape by-products can impair the digestibility of protein and amino acids through the ability of polyphenolic compounds to aggregate and precipitate proteins. One strategy for mitigating this antinutritive effect is the use of fermented grape by-products from winemaking compared with unfermented grape by-products from juice production, because the fermentation process causes an increased release of polyphenols, thereby reducing the interaction between these polyphenols and dietary protein. In contrast, the addition of exogenous tannase (tannin acyl hydrolase) was found to be ineffective in mitigating the antinutritive effect of polyphenols on the digestibility of protein in broilers.

### 3.3. Effect of Grape By-Products on Gut Morphology and Integrity

A substantial number of studies have evaluated the impact of grape by-products on gut morphology metrics and the parameters of gut integrity and permeability in fast-growing broilers, because these parameters are strongly linked with digestive and absorptive capacities, feed efficiency and systemic health, respectively.

An improvement in jejunum histology in response to feeding a basal diet with pure grape proanthocyanidins (7.5, 15, 30 mg/kg diet) for 3 and 6 weeks to Cobb 500 broilers was reported by Yang et al. [52]. After 3 and 6 weeks, the villus height:crypt depth ratio was increased (linear and quadratic effect) and the crypt depth was decreased in broilers fed the diets supplemented with grape proanthocyanidins. The strongest effects were seen at 15 mg/kg diet after 3 weeks and at 7.5 mg/kg diet after 6 weeks. Similar findings were reported in a study by Viveros et al. [56], in which the effect of two diets with different grape by-products (grape pomace concentrate, grape seed extract) but similar concentrations of extractable polyphenols in the diets (2.9 and 2.7 g/kg, respectively) was studied in male Cobb broilers. The diet with grape pomace concentrate (60 g/kg diet) and the diet with grape seed extract (7.2 g/kg diet) compared to the control diet for 3 weeks reduced the crypt depth in the jejunum mucosa. In addition, the diet with grape pomace concentrate but not with grape seed extract increased the villus height:crypt depth ratio compared to the control diet. A lengthening of the villus and a shorting of the crypt is well-known to improve nutrient absorption, decrease secretion in the gastrointestinal tract, reduce disease incidence, and increase overall performance. Thus, the reduced villus height:crypt depth ratio in broilers fed the grape seed extract could explain their poorer growth performance when compared to broilers fed the grape pomace concentrate [56]. Despite the concentrations of extractable and hydrolyzable polyphenols being similar between the two diets containing either the grape pomace concentrate or the grape seed extract, it was speculated by the authors that the presence of specific polyphenolic compounds in the grape seed extract might have caused the growth depressive effect observed. Moreover, the *Muscularis* thickness was increased in the broilers fed diets with grape pomace concentrate and grape seed extract in comparison to those fed the control diet. A beneficial increase in the villus height and villus height:crypt depth ratio in the duodenum and the jejunum was also found in Cobb 500 broilers fed a basal diet supplemented with 25 g of grape pomace per kg diet for 6 weeks compared to broilers fed the unsupplemented diet [39].

In contrast, no effect of supplementing a basal diet with a herbal extract blend (1.5 g/kg diet) consisting of grape seed extract (30%), wheat germ (20%), hop extract (25%), and inert silica carrier (25%) on the parameters of jejunal morphology, including the villus height, crypt depth, and their ratio, was found in Arbor Acres broilers [36]. The lack of effect in this study could be attributed to the relatively low dose of grape seed extract and the resulting low intake of bioactive compounds from grape seeds. Since the grape seed extract was fed as a blend with other plant components, it is also possible that the potentially beneficial actions of bioactive compounds from grape seeds were antagonized by constituents from the other blend components. In addition, no effect of feeding broilers a basal diet supplemented with a commercial white grape product on gut morphology metrics, as well as the goblet cell number and goblet cell density in the ileum and cecum, was demonstrated in two different broiler breeds (Ross 308 and Cobb 500) compared to those fed the unsupplemented basal diet [70,71]. In the latter two studies, the unaltered concentrations of microbial products in the ileum and cecum digesta indicated that the gut microbiota composition was not affected. Thus, it is likely that the lack of a shift in the microbiota composition towards more beneficial gut-barrier-protective bacteria is responsible for the unaltered gut morphology. Moreover, diets with either raw or fermented grape pomace (each 15 g/kg diet), compared to a basal diet for 6 weeks, had no effect on the villus height, crypt depth and their ratio in the ileum of Ross 308 broilers [44]. Furthermore, Chamorro et al. [69] that a basal diet supplemented with either 2.5 or 5 g/kg diet of a commercial grape extract had no effect, compared to an unsupplemented basal diet for 3 weeks, on the morphological metrics (villus height, crypt depth, goblet cell number) of the jejunum of male Cobb broilers, despite this study showing a beneficial reduction in *Escherichia coli* numbers in the ileum digesta. Since the number of lactic acid bacteria, which include beneficial *Lactobacillus* spp. and *Enterococcus* spp., was also reduced, it is possible that the positive effect of these bacteria on gut integrity and morphology did not come into play.

Apart from studies testing the isolated effect of grape by-products, two studies have studied the combined effects of grape products together with other feed additives [42,72]. As a result, these two studies have only limited significance in determining whether grape products influence gut integrity or gut morphology. In a study by Chand et al. [42], the combined effect of grape seed powder at 2.5 and 5 g/kg diet, together with organic zinc (zinc-glycine at 50 mg/kg diet), was investigated in male Hubbard broilers artificially infected with *E. tenella.* The histopathological analysis of the ceca showed the shortening of the crypt, the sloughing of villi, a glandular necrotic structure and severe hemorrhages in broilers infected with *E. tenella* only, whereas infected broilers supplemented with either dose of the grape by-product and organic zinc showed only mild sloughing of the villi and reduced hemorrhages. Although these findings show that treatment improved the disturbed gut histology of *E. tenella*-infected broilers, it cannot be determined to what extent the grape seed powder contributed to this effect. Yvon et al. [72] investigated the effect of the combined supplementation of a grape seed and skin extract (1 g/kg diet), together with specific amino acids (arginine, threonine, glutamine), for 5 weeks in Ross PM3 broilers subjected to corticosterone-induced stress. The morphological measurement of jejunum villi and crypts revealed that the reduction in villus width, crypt depth and crypt surface induced by short-term corticosterone treatment was abolished by 5 weeks of combined supplementation with the grape by-product and amino acids in broilers subjected to corticosterone treatment. The mRNA level of the protective mucin 2 (*MUC2*) gene in the jejunum mucosa was decreased by the corticosterone challenge, but combined supplementation with the grape by-product and amino acids increased the *MUC2* mRNA level after 16 days. In addition, while the concentration of the gut permeability marker fluorescein isothiocyanate dextran (FITC-d) was elevated in the blood in response to the corticosterone challenge, combined supplementation with the grape by-product and amino acids tended to decrease the FITC-d concentration in the blood of broilers subjected to corticosterone. Thus, the changes in gut morphology and gut integrity observed in broilers undergoing corticosterone treatment suggests that the combined dietary treatment exerts a protective effect on the gut barrier of broilers during corticosterone-induced stress. However, the contribution of the grape by-product to this effect is unclear.

Collectively, several studies demonstrate that grape by-products improve gut morphology, as indicated by a relative lengthening of the villi and the shortening of crypts, which is associated with a higher digestive and absorptive capacity, reduced secretion into the gut lumen, increased disease resistance, and a greater overall performance. Since improvements in gut morphology and integrity are promoted by a beneficial shift in the composition of the gut microbiota, it is likely that the effects of grape by-products on gut morphology are linked to their effects on the composition of the gut microbiota, as discussed in the following chapter.

### 3.4. Effect of Grape By-Products on Gut Microbial Community

A substantial number of studies have investigated the effect of grape by-products on the gut microbiota and/or microbial fermentation products of fast-growing broilers. Most of these studies observed either a general modulation of the gut microbial composition or an alteration in the number of selected bacterial groups (gut barrier protective bacteria, pathogenic bacteria) in response to grape by-products, which has mainly been attributed to the antimicrobial effects of polyphenolic compounds [73] but might be also caused by the prebiotic effects of indigestible carbohydrates on beneficial gut microbes, such as *Lactobacillus* spp.

Amongst these studies, a beneficial increase in the number of *Lactobacillus* bacteria in the duodenum and ceca was found in Ross 308 broilers fed a basal diet supplemented with 20 and 30 g/kg diet of a grape seed powder in comparison to broilers fed the unsupplemented basal diet [35]. No effect was seen at a supplementation level of 10 g of grape seed powder per kg diet, indicating that the concentration of indigestible carbohydrates at this dose was too low to induce a prebiotic effect. In addition, a beneficial increase in the abundance of *Lactobacillus* and *Enterococcus*, both of which can induce antiinflammatory effects and contribute to the integrity of the intestinal epithelium [74], and a decrease in the abundance of pathogenic *Dysgonomonas*, whose abundance is positively correlated with inflammation [75], in the cecum in response to grape products was reported in a study on Arbor Acres broilers [36]. In the latter study, the broilers were fed a diet supplemented with 1.5 g/kg diet of an herbal extract blend containing grape seeds and the results were compared to broilers fed the unsupplemented diet for 6 weeks [36]. Moreover, a beneficial modulation of the cecal microbiota composition was also reported for broilers supplemented with 25 g/kg diet of grape pomace, as evident from increases in the abundance of the genera *Lactobacillus* and *Bacteroides* and the phylum Bacteroidetes, as well as reductions in the abundance of the phyla Firmicutes and Proteobacteria [39]. This assumption of a beneficial modulation is based on the fact that the abundance of Proteobacteria is positively correlated with inflammatory bowel disease and is an indicator of microbial dysbiosis [76,77], while *Lactobacillus* spp. and *Bacteroides* play a key role in maintaining the intestinal barrier and exert antiinflammatory effects on the intestinal epithelium [74]. However, the concentration of microbial fermentation products in the cecum digesta was not affected, despite the effects of grape pomace on the cecal microbiota composition. This shows that measuring the concentration of microbial fermentation products in the digesta alone has only limited significance in terms of predicting changes in the gut microbial community structure. Furthermore, Gungor et al. [43] demonstrated that feeding a diet supplemented with 5 g/kg diet of *Aspergillus niger*-fermented grape seeds to female Ross 308 broilers increases the number of *Lactobacillus* spp. and decreases the prevalence of the opportunistic pathogen *Staphylococcus aureus* in the cecum digesta. However, no effect on these microbial populations was seen in this study when the diet was supplemented with 5 g of unfermented grape seeds per kg diet. The reason for only fermented grape seeds showing an effect on the number of these two microbial groups is unclear, but it is possible that certain metabolites from *Aspergillus niger* induced an antimicrobial effect on *Staphylococcus aureus*, as suggested by recent findings [73,78]. In addition, it has to kept in mind that fermentation of grape by-products causes a decrease in the concentrations of main phenolic compounds and total extractable polyphenols in the by-product, and feeding of the fermented by-product causes reduced concentrations of polyphenols in the ileal digesta when compared to feeding the unfermented by-product [46]. Thus, the antimicrobial activity against specific microbial populations is probably affected by the fermentation status of the grape by-product fed to the broilers. Although speculative, the number of *Lactobacillus* spp. could have been increased due to there being less competition between opportunistic pathogens, which had been reduced, and protective commensals (*Lactobacillus* spp.). In another study by the same group [44], feeding diets supplemented with 15 g/kg diet of either unfermented or fermented grape pomace to Ross 308 broilers did not affect the numbers of *Lactobacillus* spp. and *Staphylococcus aureus* in the cecum, but feeding them the diet supplemented with fermented grape pomace reduced the number of pathogenic *Clostridium perfringens* in the cecum digesta. The authors proposed that the latter effect was caused by the antimicrobial compounds produced by *Aspergillus niger* during the fermentation of the grape pomace. The lack of effect that fermented grape pomace has on the numbers of *Lactobacillus* spp. and *Staphylococcus aureus*, however, is unclear, considering the observations from fermented grape seeds in the study mentioned before [43]. It is possible that the metabolite spectrum resulting from the fermentation of grape seeds differs from that of grape pomace in a way that metabolites with antimicrobial activity against *Staphylococcus aureus* are less abundant in fermented grape pomace. Likewise, a beneficial effect against pathogenic bacteria was reported by Chamorro et al. [69], who showed that feeding a basal diet supplemented with 2.5 or 5 g of grape extract per kg diet to male Cobb broilers for 3 weeks caused a linear reduction in the ileal counts of *Escherichia coli*, *Enterobacteriaceae* and lactic-acid bacteria, whereas no response was observed for *Clostridium perfringens*. Similarly, feeding broilers a diet supplemented with grape seeds at different doses (10, 20 and 40 g/kg diet) for 42 days was found to decrease the ileal counts of pathogenic *Escherichia coli* and *Streptococcus* spp. but to increase the ileal count of beneficial *Lactobacillus* spp. in Cobb 500 broilers [50]. Furthermore, feeding a diet supplemented with a grape seed extract (0.2 and 0.4 g/kg diet) to Arbor Acres Plus broilers caused an increase in the abundance of beneficial bacterial genera, such as *Lactobacillus*, as assessed by LEfSe analysis [45]. Partially beneficial effects were reported by Viveros et al. [56], in which the number of beneficial *Lactobacillus* spp. and *Enterococcus* spp. but also the number of pathogenic *Escherichia coli* in the cecum content were increased in male Cobb broilers fed a basal diet supplemented with either grape pomace concentrate (60 g/kg diet) or grape seed extract (7.2 g/kg diet). This study indicated that grape by-products simultaneously promote the growth of both beneficial and detrimental bacteria, but obviously the balance between populations of beneficial and pathogenic bacteria was not impaired, as the authors stated. Contradictory to these findings in the cecum, the same study revealed, regarding the ileum content, that the number of *Lactobacillus* was reduced by the grape pomace concentrate diet, that the number of *Enterococcus* was increased by the grape seed extract diet and that the number of *Escherichia coli* was not altered by both grape by-product diets [56]. Although the reason for these contradictory findings is unclear, this shows that the effects of grape by-products on the gut microbial composition is also dependent on the gut segment studied. One explanation for this could be that the concentrations of phenolic compounds with antimicrobial activity in the digesta decrease during passage from the small to the large intestine. Regarding the partially discrepant results for the two grape by-products in the ileum microbiota, it has to mentioned that the concentrations of the extractable polyphenols in the two diets were similar (2.9 and 2.7 g/kg, respectively). Consequently, it is more likely that the discrepant results were caused by different concentrations of indigestible carbohydrates between the grape pomace concentrate diet and the grape seed extract diet.

Supplementation with grape by-products had no effects on the gut microbiome in a few studies. In a study on male Cobb broilers fed diets with either unfermented or fermented grape skins at two different doses (30 and 60 g/kg diet) for 3 weeks, supplementation had no effect on the counts of lactic acid-bacteria and *Clostridium* spp. in the ileum, whereas the number of *Escherichia coli* in the ileum was higher in the broilers fed the fermented diet than in those fed the unfermented grape skins [46]. A possible explanation for this is that the higher levels of polyphenols observed in unfermented grape skins exerted a growth-suppressing effect on *Escherichia coli* in the ileum. In agreement with this, higher concentrations of total extractable polyphenols were found in the ileal digesta of broilers fed the unfermented grape skins [46]. In addition, in two studies by Duangnumsawang et al. [70,71], a basal diet supplemented with a grape extract had no effect on the concentrations of diverse bacterial metabolites in the ileum and cecum of fast-growing broiler breeds (Ross 308 and Cobb 500) compared to those fed an unsupplemented diet. Although the microbiota composition in the ileum and cecum was not analyzed in these studies, the observation that bacterial metabolic activity was obviously not altered could indicate that the microbiota community structure was not modulated by the grape extract. An explanation for the lack of effect on the ileal and cecum microbiome in these two studies [70,71] could be the low concentration of total polyphenols in the diet supplemented with the grape extract when compared to other studies [46]. Moreover, a further study reporting only the concentrations of microbial fermentation products showed that a diet supplemented with 10 g/kg diet of a grape seed extract had no impact on the concentrations of total short-chain fatty acids and the proportions of individual short-chain fatty acids in the cecal digesta [55]. In this study, the concentration of total polyphenols in the diet supplemented with grape seed extract was also comparatively low, which might also serve as an explanation for the lack of effect. In addition, the relatively young age of the broilers (21 days), which was lower than that in almost all other studies reporting an effect on the gut microbiome, might have also contributed to the lack of effect.

Apart from the above-presented studies, two studies [57,79] investigated the effect of grape products in attenuating artificially induced coccidiosis. In a study by Wang et al. [57], it was shown that the number of *E. tenella* oocysts following infection with *E. tenella* decreased in broilers fed diets with either 5, 10, 20, 40 and 80 mg/kg diet of an ethanolic grape seed extract compared to infected control broilers fed the same diet without the grape product. Treatment with the grape product was as effective as the treatment of a further group of infected broilers with the antibiotic salinomycin. In contrast, a study by Naidoo et al. [79] observed no reduction in oocyte output in the feces of *E. tenella*-infected broilers receiving an ethanolic grape seed extract via gavage (75 mg/kg BW) once a day for 5 consecutive days compared to infected broilers receiving no grape seed extract.

**Figure 1:**
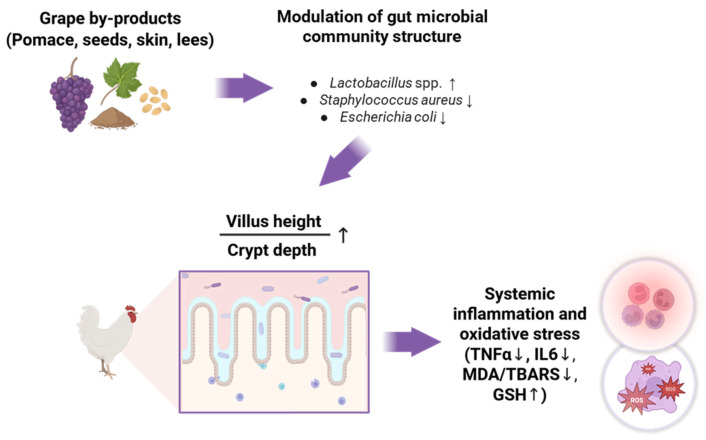
Scheme showing the link between the modulation of the gut microbiota, gut morphology and systemic inflammation and oxidative stress in fast-growing broilers. Feeding grape by-products to fast-growing broilers beneficially modulates the gut microbial composition by increasing the numbers of beneficial commensal bacteria, such as *Lactobacillus* spp., while reducing those of pathogenic bacteria (e.g., *Staphylococcus aureus*). Commensal gut bacteria strongly influence intestinal epithelial cell physiology and play a crucial role in maintaining gut morphology and the homeostasis of the intestinal barrier. In contrast, a perturbation of the commensal gut microbiota by pathogenic bacteria—referred to as gut dysbiosis—triggers an inflammatory response in intestinal epithelial cells, increases apoptosis, and leads to the shedding of intestinal epithelial cells at the villus tip, resulting in villus shortening (villus atrophy). Consequently, gut dysbiosis weakens the gut barrier, causing intestinal hyperpermeability and an increased translocation of bacterial endotoxins, which induce systemic inflammation and oxidative stress. Thus, preventing gut dysbiosis by feeding broilers grape by-products supports improved gut integrity and morphology while reducing systemic inflammation and oxidative stress. ↑, increase; ↓, decrease. Created in BioRender. Eder, K. (2025) https://BioRender.com/jln80i6 (accessed on 20 April 2025).

To sum up, feeding grape by-products to fast-growing broilers shows a positive effect on the gut microbial composition (increased number of beneficial bacteria, reduced numbers of pathogenic bacteria) in the vast majority of studies. These effects are likely the net result of the antimicrobial activities of phenolic compounds and the prebiotic actions of indigestible carbohydrates. It is well-known that the presence of beneficial bacteria, such as *Lactobacillus* spp. and *Bifidobacterium* spp., in the gut contributes to improved gut integrity and gut morphology, as indicated by an increased villus height:crypt depth ratio; meanwhile, pathogenic bacteria have the opposite effect (Figure 1). Since the lengthening of the villi and shortening of the crypts are associated with a higher digestive and absorptive capacity and a greater overall performance, it can be assumed that the improved gut morphology observed in studies with broilers fed different grape by-products [39,52,56] is linked to the beneficial shift in the gut microbiota composition.

### 3.5. Effect of Grape By-Products on Oxidative Stress and Antioxidant Status

Owing to the well-known antioxidant action of polyphenols, it is not surprising that a large number of feeding studies have studied the effect of grape by-products on oxidative stress and antioxidant status in fast-growing broilers. With only a few exceptions, the vast majority of studies showed a reduction in oxidative stress parameters, such as the levels of malondialdehyde (MDA) or thiobarbituric acid-reactive substances (TBARS), or an improvement in antioxidant indices, e.g., total antioxidant activity, activity of antioxidant enzymes [glutathione peroxidase (GPX), superoxide dismutase (SOD), catalase (CAT), glutathione-S-transferase (GST), glutathione reductase (GR)], the concentration of antioxidants like total or reduced glutathione (GSH) and vitamin E, in plasma or serum and different tissues by feeding grape by-products. For instance, Ross 308 broilers fed a basal diet containing oxidized rice bran oil to induce oxidative stress and supplemented with 100 mg of grape seed extract per kg diet had decreased MDA levels and an increased total antioxidant capacity and GPX activity in their serum and liver, and elevated hepatic mRNA levels of the redox-sensitive transcription factor nuclear Factor Erythroid 2-Related Factor 2 (*NRF2*), *CAT* and hemeoxygenase 1 (*HO1*) compared to broilers fed the basal diet containing oxidized rice bran oil without grape seed extract [33]. An increase in plasma GSH-Px activity and a decrease in the plasma concentration of MDA was also reported for Ross 308 broilers fed diets with 20 and 30 g/kg diet of a grape seed powder for 42 days compared to broilers fed the diet with 0 and 10 g/kg diet of grape seed powder [35]. Another study demonstrated that feeding diets supplemented with 50 and 100 g/kg diet of red grape pomace to Cobb broilers for 3 weeks increased their plasma concentrations of α-tocopherol and Trolox equivalents [63]. In addition, a dose-dependent increase in the activity of antioxidant enzymes (SOD, CAT, GPX, GST) and GSH and a decrease in the concentration of TBARS in blood was observed in Cobb 500 broilers fed a diet supplemented with graded levels of grape seeds compared to broilers fed the unsupplemented basal diet [50]. Furthermore, Makri et al. [80] demonstrated that feeding a diet supplemented with red grape pomace to female Hubbard broilers for 15 and 35 days increased the concentration of GSH in erythrocytes and the total antioxidant capacity in plasma and decreased the concentration of TBARS compared to broilers fed the control diet. Moreover, in a study with Ross 308 broilers vaccinated against the Newcastle disease virus, the supplementation of broiler chicken diets with a grape seed extract at different concentrations (0.125, 0.25, 0.5, 1 and 2 g/kg diet) increased the concentration of reduced GSH and decreased that of MDA in the liver, whereby even the lowest concentration was effective and higher concentrations did not improve the effect [51]. Adding a grape seed extract to the diet (12 mg/kg diet) increased plasma SOD activity but did not decrease the plasma MDA concentration in broilers infected with *E. tenella* in comparison to infected broilers fed a control diet [57]. A study by Yang et al. [52] revealed that feeding a diet supplemented with grape proanthocyanidins to Cobb 500 broilers for 6 weeks increased the SOD concentration and decreased the MDA concentration in plasma at 7.5 and/or 15 mg/kg diet in comparison to broilers fed the unsupplemented diet. At a concentration of 30 mg/kg diet, the grape proanthocyanidins had no effect on these antioxidant indices when compared to broilers fed the unsupplemented diet. In a further study with Ross 308 broilers, only a partial beneficial outcome was found, as the activity of SOD in plasma was increased but the concentration of MDA, ferric reducing antioxidant power (FRAP) and the activities of CAT and GPX in the plasma were not affected by a diet supplemented with 2 g of an olive leaf and grape-based by-product per kg diet compared to the unsupplemented diet for 40 days [37]. Using RNA-seq, feeding a diet supplemented with 2 g of grape pomace extract per kg diet to Ross 308 broilers was demonstrated to increase the jejunal expression of several redox-sensitive genes, all of which were involved in antioxidant defense, compared to broilers fed the unsupplemented diet for 45 days [38]. However, the concentration of TBARS in breast muscle was not affected by dietary treatment. An increase in the activity of GPX and CAT in the serum was reported for Ross 308 broilers fed a diet supplemented with unfermented grape seeds (5 g/kg diet) [43]. In contrast, the diet supplemented with fermented grape seeds (5 g/kg diet) did not increase the activities of these antioxidant enzymes in comparison to broilers fed the unsupplemented diet for 42 days [43]. Feeding a diet supplemented with either wine lees extract (2 g/kg diet) or grape stem extract (0.1 g/kg diet) to Ross 308 broilers for 6 weeks was reported to increase the activity of SOD in the plasma [40]. The plasma activities of other antioxidant enzymes, such as CAT, GPX, GST and GR, and the plasma concentrations of the oxidative stress indicator MDA or different antioxidant capacity measures like FRAP, 2,2′-Azinobis-(3-Ethylbenzthiazolin-6-Sulfonic Acid (ABTS) or 2,2-diphenyl-1-picrylhydrazyl (DPPH) were not altered by these grape by-products [40]. In breast muscle, the diet with wine lees extract and grape stem extract decreased the MDA concentration in breast muscle and the diet with grape stem extract increased the antioxidant capacity indicator FRAP in breast muscle. The mRNA levels of important genes involved in the antioxidant system, such as *CAT*, *GPX1*, *GPX2*, *SOD1* and *GST2A*, in the liver were not affected by any of the dietary treatments in this study [40]. Curiously, the diet supplemented with ground grape pomace (25 g/kg diet) had no effect on either of these parameters in the plasma and breast muscle, despite the concentrations of total polyphenolic compounds in this diet being markedly higher than in the diets supplemented with wine lees extract or grape stem extract. The authors of this study did not provide an explanation for this observation, but it is possible that the lack of effect had by grape pomace was due to the fact that condensed tannins, such as procyanidin B1 and procyanidin B2, significantly contributed to the high concentration of polyphenolic compounds but that the bioavailability of condensed tannins was rather low.

Apart from the above-described studies exploring the effect of grape by-products on oxidative stress and the antioxidant status of broilers under normal (healthy) conditions, two studies on Cobb broilers by Rajput et al. [49,81] investigated the effect of grape by-products under the conditions of diet-induced aflatoxicosis, which is known to impair the antioxidant defense system and cause oxidative stress. In line with this, an aflatoxin B1-contamined diet caused a downregulation of hepatic mRNA and a decrease in the protein levels of the antioxidative key regulator NRF2 and its target genes including HO1 and GPX1; meanwhile, supplementation of the aflatoxin B1-contaminated diet with 0.25 g of a grape seed extract per kg diet increased the mRNA and protein levels of NRF2 and its target genes [81]. In another study by the same group [49], an aflatoxin B1-contamined diet supplemented with a grape seed extract at two doses (0.25 and 0.5 g/kg diet) counter-regulated the increase in the serum MDA concentration and decreased the serum activities of antioxidant enzymes in broilers fed the aflatoxin B1-contaminated diet without the grape product. These findings clearly show that grape by-products protect from aflatoxicosis-induced oxidative stress in broilers.

Only two studies reported either no effects or inconsistent effects on oxidative stress and antioxidant status. No effect of dietary supplementation with either 0.1 or 0.2 g of grape seed extract per kg diet for 6 weeks on the plasma concentration of TBARS was observed in Ross 308 broilers in comparison to broilers fed a control diet [82]. One explanation for the lack of effect in the latter study might be the relatively low dose used when compared to studies with a positive outcome, as described above. Inconsistent findings were reported in a study of Fejerčáková et al. [53], in which the 6-week administration of a plant extract containing red grape pomace via drinking water decreased SOD and GPX activities in the liver and heart mitochondria but increased SOD activity in the plasma and kidney of Cobb 500 broilers. In addition, the concentration of GSH was increased in broilers administered the plant extract but not in the liver, heart and kidney. The partially contradictory effects of grape pomace on the activity of the same antioxidant enzyme (SOD) in different tissues, however, cannot be explained without speculation.

Several of the studies investigating the antioxidant effects of dietary grape by-products in fast-growing broilers also focused on the impact of dietary treatments on the oxidative stability of broiler meat. In this regard, Romero et al. [41] revealed that feeding a diet supplemented with grape seeds (24.4 g/kg) and grape skins (13.1 g/kg) to Cobb broilers for 3 weeks elevated the plasma concentrations of α- and γ-tocopherol compared to the unsupplemented diet. In addition, the concentrations of α- and γ-tocopherol in thigh meat after 7 days of refrigerated storage were increased, whereas the TBARS concentration in thigh meat after 7 days of refrigerated storage was decreased in broilers fed the diet supplemented with grape seeds and grape skins. Likewise, the inclusion of grape pomace concentrate in the diets at 30 and 60 g/kg diet enhanced the serum antioxidant activity, as determined by the ABTS method, and reduced the MDA values in breast samples after 1, 4 and 7 days of refrigerated storage compared with samples obtained from male Cobb broilers fed the control diet [58]. A study from Turcu et al. [83] demonstrated that feeding a diet with either 30 or 60 g of white grape pomace or 30 or 60 g of red grape pomace per kg diet for 42 days to Cobb 500 broilers reduced the concentration of TBARS in thigh meat in comparison to broilers fed the unsupplemented diet. In breast meat, the concentration of TBARS was only reduced in broilers fed the diet supplemented with 30 g of white grape pomace per kg diet and that supplemented with 60 g of red grape pomace per kg diet. Other lipid oxidation parameters, such as conjugates dienes and trienes and the *p*-anisidine value in thigh and breast meat, were not affected by dietary treatments. In addition, the concentration of TBARS linearly decreased in the breast meat of Ross 308 broilers fed diets with increasing concentrations of grape pomace (5, 7.5 and 10 g/kg diet) after 0, 5 and 10 days of storage at −4 °C [48]. This finding indicated that dietary grape pomace improves the oxidative stability of broiler meat during storage. Likewise, breast meat stored at 4°C for 3 and 7 days from Ross 308 broilers fed diets with 50 and 70 g of grape pomace per kg diet had a lower MDA concentration than that from broilers fed either the unsupplemented diet or the diet supplemented with 25 g of grape pomace per kg diet [84]. In line with an improvement in the oxidative stability of the breast meat, the concentration of hexanal, an established marker of lipid oxidation in food, was reduced in meat from broilers fed the grape-pomace-supplemented diets. The concentration of TBARS in thigh meat after 7 days of refrigerated storage was also shown to be reduced by feeding diets supplemented with two doses (30 and 60 g/kg) of either unfermented or fermented grape skins to Cobb broilers for 3 weeks in comparison to the unsupplemented diet [46]. In a second study by Gungor et al. [44], in which the same grape products were fed to Ross 308 broilers for 42 days at a higher dose (each 15 g/kg diet) than in the study before [43], the concentration of MDA in breast meat after 45 min and 11 days of storage was not affected by either fermented or unfermented grape pomace. This lack of effect is hard to explain because the unfermented grape pomace was found to improve the antioxidant defense capacity, as is evident from the elevated plasma activities of GPX and SOD [44].

Based on the studies cited, it can be concluded that using grape products across a wide dosage range is an effective strategy for enhancing the antioxidant status and protecting against oxidative stress in broiler tissues, and improving the oxidative stability of broiler meat. Although TBARS have been used in most studies—and as the only parameter in some studies—as an indicator of oxidative stress, the TBARS assay should be generally considered critically due to several limitations (e.g., the low specificity of TBARS, which react with various substrates to form MDA, and the fact that large amounts of TBARS are formed during the assay itself). In contrast, a more specific marker of lipid oxidation is cholesterol oxides, such as 7β-hydroxycholesterol and 7-ketocholesterol, which are also suitable for evaluating the oxidative stability of broiler meat [85]. Thus, future studies exploring the effects of grape by-products on the antioxidant status and the oxidative stability of meat should not only rely on TBARS levels but measure a panel of different biomarkers including the above-described (e.g., antioxidant enzyme activities, vitamin E) and some specific parameters of lipid oxidation, such as cholesterol oxides.

### 3.6. Effect of Grape By-Products on Immune Response

Several studies have addressed the effects of grape by-products on the immune response in fast-growing broilers, with the effects being evaluated both under normal (without challenge) and immune challenge conditions (e.g., aflatoxicosis, vaccination, corticosterone treatment).

Under normal conditions, a stimulatory effect on the mucosal immune response was reported for Ross 308 broilers fed a grape-product-supplemented diet (2 g/kg diet) for 15 and 35 days. This was evident from a higher density of intraepithelial leukocytes, especially CD45+, and the regulation of a number of genes involved in the inflammatory response and antimicrobial responses in the jejunum mucosa [38]. However, the diet with the grape product caused the regulation of both proinflammatory (e.g., *C1QTNF44* and *CXCL12*) and antiinflammatory genes (e.g., *BD9* and *BD10*). Thus, the net effect of feeding the grape product on the inflammatory response cannot be evaluated from this study. In contrast, the antiinflammatory effect of feeding an aqueous grape seed extract (0.2 or 0.4 g per kg diet) to Arbor Acres Plus broilers for 3 weeks in the absence of an immune challenge was reported by Cao et al. [45]. In this study, broilers fed the diet supplemented with the lower dose of grape seed extract had lower serum concentrations of proinflammatory cytokines (IL6, IL1β) when compared to broilers fed the unsupplemented control diet. In addition, the concentration of IL1β in the ileal and jejunal mucosal was lower in broilers fed the grape-seed-extract-supplemented diets (low and high dose) than in the control broilers. Furthermore, feeding broilers grape-seed-extract-supplemented diets (low and high dose) tended to decrease the IL8 concentration in the jejunum mucosa and feeding broilers the diet with a high dose of grape seed extract tended to decrease the IL6 concentration in the ileum mucosa. Finally, the serum levels of the immunoglobulins IgA and IgY were shown to be increased in broilers fed the low dose and the high dose of the grape seed extract, respectively, compared to broilers fed the control diet [45]. Based on additional results from gut microbiome analyses, the authors speculated that the grape seed extract may have changed the mucosal immune system at the jejunum and ileum surfaces by regulating the gut microbiota. No effect of feeding a wheat–soybean meal diet supplemented with a commercial white grape product to male and female Ross 308 and Cobb 500 broilers in the absence of an immune challenge on the expression of a large set of inflammatory genes (*IL1B*, *IL2*, *IL4*, *IL6*, *IL8*, *IL10*, *IL12*, *IL17A*, *IL18*, *IFNG*, *TGFB2*) in the ileum mucosa was found [71]. Likewise, in another study of the same group, almost no effect of feeding the white grape product to male and female Ross 308 and Cobb 500 broilers not exposed to an immune challenge on the expression of the same inflammatory genes in the cecum mucosa was seen [70]. The only effect seen was a higher expression of *IL10* in broilers fed the diet supplemented with the grape product. Considering that the cytokine IL10 plays an important antiinflammatory role [86], the grape product can be considered beneficial. One study observed the divergent effects of feeding diets supplemented with different types and doses of grape by-products (25 g grape pomace/kg diet, 2 g wine lees extract/kg diet or 1 g grape stem extract/kg diet) to Ross 308 broilers for 42 days in the absence of an immune challenge [87]. Although the contents of total phenolic, total flavonoid and total tannin compounds markedly differed between the three diets containing the different grape by-products, the mRNA levels of most proinflammatory genes investigated (*TNFA*, *INFA*, *INFG*, *IL1B*, *IL18*) in the liver were not affected compared to the unsupplemented diet [87]. However, in the spleen and the Bursa of Fabricius, the diet with the grape stem extract, which had a significantly lower content of phenolic, flavonoid and tannin compounds than the diet with grape pomace [40], caused an upregulation of *IL8* and *TLR4* compared to the control diet, whereas the diet supplemented with grape pomace had no effect. These findings indicate that the effect of grape by-products on the immune response is dependent on the tissue investigated and the type of grape by-product, which largely differ in their content and type of polyphenolic compounds (e.g., grape pomace vs. grape stems [40]). A further study that was performed with broilers in the absence of an immune challenge has only limited significance, because the grape product was not administered alone. In the latter study, the effect of the combined administration of red grape pomace (30 g/kg diet) and Aloe vera gel (provided via the drinking water at 1, 2, 3, and 4%) compared to a control diet without red grape pomace and no provision of Aloe vera gel for 42 days on different blood parameters was investigated in Ross 308 broilers [34]. Despite a positive quadratic effect on the count of basophils—a type of white blood cell involved in the immune response and inflammatory processes—with an increasing dose of Aloe vera in the drinking water, the combined administration of red grape pomace and Aloe vera gel had no effect on any immune-related parameters.

In contrast to the less consistent effects in broilers without a specific immune challenge, the effects of grape by-products on immune function were clearer in different immune challenge models. For instance, the beneficial immunostimulatory effect of a grape seed extract to Cobb broilers was found under conditions of feeding-induced aflatoxicosis [49]. While feeding a basal diet containing aflatoxin B1 (1 mg/kg diet) reduced serum levels of IgA, IgG, and IgM compared to the basal diet without aflatoxin B1, which is in line with the known immunosuppressive effect of aflatoxin B1 [88], the addition of a commercial grape seed extract (0.25 or 0.5 g/kg) to the aflatoxin B1-contaminated basal diet increased the serum levels of all immunoglobulins. This finding suggested that the grape seed extract counteracts the immunosuppressive effect of aflatoxin B1 on the humoral immune system of broilers. The uncontaminated basal diet supplemented with 250 mg/kg diet of the grape product had no effect on the serum levels of IgA, IgG, and IgM compared to the uncontaminated basal diet, indicating that the grape by-product is ineffective under “healthy” conditions. The authors concluded that the dietary supplementation of grape seed extract (0.25 and 0.5 g/kg) promotes immune function under conditions of aflatoxicosis, which is known to impair growth performance and immunity in broilers. Based on their findings, the same authors investigated if the grape seed extract is able to protect the spleen from aflatoxin B1-induced immune injury by suppressing the inflammatory response and inhibiting NF-κB expression in broilers [81]. In fact, this study showed that broilers fed the aflatoxin B1-contaminated diet (1 mg/kg diet) supplemented with grape seed extract (0.25 g/kg diet) had lower mRNA levels of proinflammatory cytokines (TNFα, IFNγ, IL1β, and IL6) in the spleen and lower serum concentrations of these cytokines than broilers fed the contaminated diet alone. In addition, the grape-seed-extract-supplemented aflatoxin B1-contaminated diet normalized NF-κB p65 phosphorylation and IκBα degradation induced by feeding the aflatoxin B1-contaminated diet. These findings indicated that grape seed extract protects broilers from aflatoxin B1-induced immunotoxicity by inhibiting the NF-κB-driven inflammatory response. The stimulatory effect of a grape by-product (aqueous grape seed extract) on the humoral immune response was also reported in a study with Ross 308 broilers under conditions of vaccine-induced immune activation [51]. In this study, the effect of dietary supplementation with an aqueous grape seed extract at levels of 0.125, 0.25, 0.5, 1 and 2 g/kg diet on the humoral immune response against Newcastle disease virus vaccine was investigated. Diets with the grape seed extract resulted in a significant increase in the specific antibody titer against Newcastle disease virus vaccine at 28 and 35 days of age, with the highest antibody titer found at the dose of 0.5 g grape seed extract/kg diet compared to the basal control diet. These findings suggest that grape seed extract might serve as a natural immunostimulatory agent in broilers to provide immune protection against Newcastle disease virus. Inconclusive results regarding the effects of the combined supplementation of a grape seed and skin extract (1 g/kg diet) with specific amino acids (arginine, threonine, glutamine) were reported in a study with Ross broilers under corticosterone-induced stress [72]. While the treatment of broilers with corticosterone alone tended to decrease the gene expression of the proinflammatory gene *TNFA* in the jejunum mucosa, which is in line with the immunosuppressive effect of corticosterone in broilers [89], *TNFA* was surprisingly upregulated by the combined supplementation of the grape seed and skin extract and specific amino acids during corticosterone treatment after 16 days. In contrast, after 35 days of treatment, no upregulation of *TNFA* in the jejunum mucosa was seen in corticosterone-treated broilers receiving the grape seed and skin extract together with amino acids. Considering the latter and the observation that the expression of other inflammatory genes (*IFNG*, *IL10*) was not affected by the combined treatment with grape seed and skin extract and amino acids, these findings suggest that grape polyphenols do not modulate the intestinal immune response of broilers subjected to corticosterone treatment.

Overall, it can be summarized that some studies show the beneficial effect of grape by-products on the immune response of broilers, particularly under conditions of immune challenge. Less consistent effects, as observed in broiler studies in the absence of a specific immune challenge, cannot be solely ascribed to the use of different types and doses of grape by-products, but to heterogenous experimental designs with differences in the outcome criteria (e.g., immunoglobulin levels, mRNA levels of inflammatory genes, cytokine levels) and analyzed tissues (intestinal mucosa, liver, spleen, plasma). In order to improve the comparability between independent studies, it is important in the future to use more standardized immunological assays, similar parameters, the same biological materials and more challenge tests (e.g., Newcastle disease virus, *Escherichia coli*).

## 4. Conclusions and Future Perspectives

**Figure 2:**
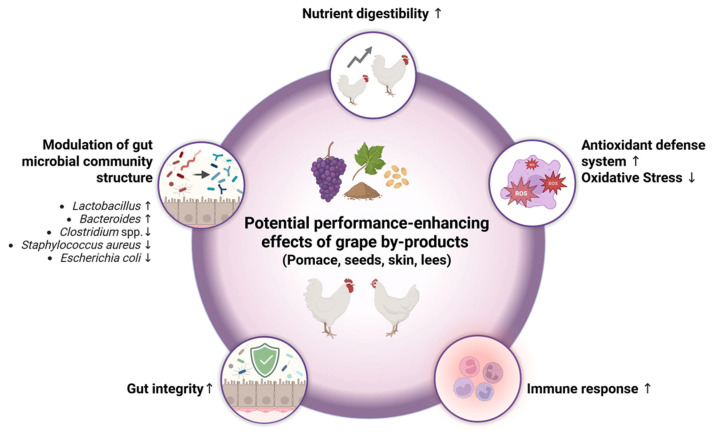
Overview of the mechanisms underlying the performance-enhancing effects of grape by-products in fast-growing broilers. The performance-enhancing effects of grape by-products in fast-growing broiler breeds can be attributed to various mechanisms such as improved nutrient digestibility, a positive influence on intestinal morphology and integrity, the favorable modulation of the microbial community in the gut, the inhibition of oxidative stress or the enhancement of the antioxidant defense system, and the stimulation of the immune response. Studies investigating the modulation of the microbial community in the gut of fast-growing broilers mostly show an increase in bacteria of the genera *Lactobacillus* and *Bacteroides*, both of which are known to be antiinflammatory and gut-barrier-strengthening bacterial groups, while the number or proportion of obligate pathogenic bacterial species such as *Escherichia coli* or *Clostridium perfringens*, which impair intestinal integrity, are reduced by grape by-products. ↑, increase; ↓, decrease. Created in BioRender. Eder, K. (2025) https://biorender.com/a46r9ia (accessed on 20 April 2025).

By-products from winemaking, such as grape pomace, grape seeds, grape skins, or extracts made from them, represent a cost-effective and sustainable bioresource. These by-products are a source of polyphenolic compounds, plant fibers and—in the case of seeds—essential fatty acids with various health-promoting effects for livestock. Numerous studies involving fast-growing broiler breeds—which often suffer from metabolic inflammation and oxidative stress due to disproportionate breast muscle growth, leading to issues like cardiorespiratory insufficiency [23,24]—indicate that supplementing feed with grape by-products improves performance, particularly weight gain and feed efficiency. This literature review demonstrates that the performance-enhancing effects of grape by-products in fast-growing broiler breeds can be attributed to various mechanisms, such as improved nutrient digestibility, a positive influence on intestinal morphology and integrity, the favorable modulation of the microbial community in the gut, the inhibition of oxidative stress or the enhancement of the antioxidant defense system, and the stimulation of the immune response (Figure 2). Studies investigating the modulation of the microbial community in the gut of fast-growing broilers mostly show an increase in bacteria of the genera *Lactobacillus* and *Bacteroides*, both of which are known to be antiinflammatory and gut-barrier-strengthening bacterial groups, while the number or proportion of obligate pathogenic bacterial species such as *Escherichia coli* or *Clostridium perfringens*, which impair intestinal integrity, are reduced by the feeding of grape by-products. Given the central importance of intact intestinal barrier function for metabolism and health [90], it can be assumed that the observed influence on the structure of the microbial community in the gut plays a significant role in improving performance.

Among the winemaking by-products, grape seeds and their extracts have proven especially effective as performance enhancers. Optimal dosages range from 5 to 30 g/kg diet for grape seeds and from 0.01 to 0.5 g/kg diet for grape seed extracts. Higher doses of grape seeds (>40 g/kg diet) or extracts (>0.5 g/kg diet) showed either no improvements or negative effects on growth performance. Therefore, when using these by-products for fast-growing broilers, dosages should not exceed 30 g/kg diet for grape seeds or 0.5 g/kg diet for grape seed extracts. Grape pomace, on the other hand, was found effective for enhancing BW gain in most studies when used in doses between 5 to 30 g/kg diet. Higher doses (≥60 g/kg diet) of grape pomace even worsened the performance of fast-growing broilers. Grape skins were found to be ineffective at a dose of 30 g/kg diet, while feeding higher doses of grape skins (60 g/kg diet) negatively affected growth performance in fast-growing broilers. Based on the studies considered in this review, the effective dosage ranges for the performance enhancement and performance inhibition of various grape by-products in feed for broilers are specified in Table 3.

However, it was observed that pretreating grape pomace with polyethylene glycol—known to bind tannins and neutralize their effects—eliminated the negative impact of high doses [47]. This indicates that at higher dosages, the antinutritional properties of grape by-products, which are likely caused by tannins, non-starch polysaccharides and/or phytic acid, may outweigh the positive effects of health-promoting polyphenolic compounds. The use of feed enzymes such as non-starch polysaccharides-degrading enzymes (e.g., cellulases, β-glucanases, xylanases, pectinases) or phytases in diets containing grape by-products for broilers could be an effective strategy for mitigating the performance-reducing effects of tannins, non-starch polysaccharides, or phytic acid while simultaneously enhancing the health-promoting effects of low-molecular-weight polyphenolic compounds found in grape by-products. This could also enable the inclusion of higher levels of grape by-products in diets for fast-growing broiler breeds, thereby improving the resource-efficiency and sustainability of broiler meat production. The findings of a study by Gungor et al. [44] suggesting that a diet supplemented with fermented grape pomace, but not with unfermented grape pomace, increases the growth performance of Ross 308 broilers supports this assumption. In this study, the grape pomace was fermented using *Aspergillus niger*, which produces a range of enzymes such as cellulases, xylanases, amylases, proteases, lipases, and even tannases [91], thereby reducing the levels of antinutritive compounds present in agri-industrial by-products [92]. Pre-fermentation with *Aspergillus niger* was thus shown to enhance the antioxidant and antimicrobial effects of plant extracts, as demonstrated in the case of a *Magnolia officinalis* extract [93].

A large body of evidence showing the positive effects of grape by-products in other livestock species, such as pigs and cattle, exists [94]. This indicates that grape by-products are generally able to improve the performance and/or health of livestock. In addition, the use of grape by-products as a feed component, which is not restricted by specific feed regulations in the EU as long as general feed safety guidelines are considered, significantly contributes to the recycling of agricultural waste from winemaking—an inexpensive and readily available resource in winemaking regions. This, in turn, enhances the sustainability of livestock production and mitigates environmental issues. Despite the advantages associated with the use of grape by-products as animal feed, future studies must also address potential drawbacks or risks, such as those arising from the application of pesticides in viticulture, which may impact animal health, the quality of animal products, and consumer health. Furthermore, future research should determine whether grape by-products demonstrate positive efficacy under practical conditions with typical stress scenarios (e.g., high stocking density, heat stress). Additionally, future studies should examine whether grape by-products, in combination with other feed additives such as probiotics or enzymes, exert synergistic or additive effects on animal performance and health.

## Figures and Tables

**Table 1:** Overview of feeding studies reporting effects of grape by-products on performance of fast-growing broilers.

Broiler Breed and Sex	Grape By-Product	Dose (g/kg Diet)	Polyphenol Content (per kg Diet)	Duration (Days)	Main Effects	Ref.
Ross 308, male	GS extract	0.1	-	42	1–42 d of age: FI: ↑, BW gain: ↑, F:G ratio: ↓	[33]
Ross 308, male	GP	30	-	28	14–42 d of age: FI: -, BW gain: ↑, F:G ratio: ↓	[34]
Ross 308, unsexed	GS	10, 20 or 30	-	42	22–42 d of age: FI: ↑ (20 and 30 g/kg diet), BW gain: ↑ (20 and 30 g/kg diet), F:G ratio: -	[35]
Arbor acres, unsexed	Herbal extract blend	1.5	-	42	1–42 d of age: FI: -, BW gain: ↑, F:G ratio: ↓	[36]
Ross 308, male	Olive leaf (62%) and grape- based by-product (24%)	2	GAE: 14 mg	40	1–40 d of age: FI: -, BW gain: -, F:G ratio: -	[37]
Ross 308, mixed sex	GP extract	2	GAE: 154 mg	44	1–44 d of age: FI: -, BW gain: ↑, F:G ratio: -	[38]
Cobb-500, mixed sex	GP	25	GAE: 2.18, 1.95 and 2.08 g in starter, grower and finisher diets	42	1–42 d of age: FI: -, BW gain: ↑, F:G ratio: -	[39]
Ross 308, unsexed	GP, wine lees extract (WLE) or grape stem extract (GSE)	25 (GP), 2 (WLE) or 0.1 (GSE)	GAE: 193 mg (GP), 7.3 mg (WLE) and 10 mg (GSE)	42	GP, WLE, and GSE: 1–42 d of age: FI: -, BW gain: -, F:G ratio: -	[40]
Cobb 500, male	GS or GK	30 (GS) or 110 (GK)	TEP: 4.09 g (GS) and 4.18 g (GK)	21	GS: 1–21 d of age: FI: -, BW gain: -, F:G ratio: -; GK: 1–21 d of age: FI: -, BW gain: ↓, F:G ratio: ↑	[41]
Hubbard, male	GS	2.5 or 5 (together with organic zinc)	-	35	8–35 d of age: FI: ↑, BW gain: ↓, F:G ratio: ↓	[42]
Ross 308, female	Fermented GP (FGP) or unfermented GP (GP)	5	Total phenolic compounds: 142.5 µg (FGP) and 117 µg (GP)	42	FGP and GP: 1–42 d of age: FI: -, BW gain: ↑, F:G ratio: -	[43]
Ross 308, male	Fermented GP (FGP) or unfermented GP (GP)	15	-	42	FGP: 1–42 d of age: FI: -, BW gain: ↑, F:G ratio: -; GP: 1–42 d of age: FI: -, BW gain: -, F:G ratio: -	[44]
Arbor Acres Plus	GS extract	0.2 or 0.4	-	21	0.2 g/kg diet: 1–21 d of age: FI: ↓, BW gain: ↑, F:G ratio: ↓; 0.4 g/kg diet: 1–21 d of age: FI: ↓, BW gain: -, F:G ratio: ↓	[45]
Cobb 500, male	Fermented (FS) or unfermented (UF) GK	30 or 60	TEP: 1.98 g (FS30), 2.27 g (FS60), 2.42 g (UF30) and 3.13 g (UF60)	21	FS30: 1–21 d of age: FI: -, BW gain: -, F:G ratio: -; FS60: 1–21 d of age: FI: -, BW gain: ↓, F:G ratio: ↑; UF30: 1–21 d of age: FI: -, BW gain: -, F:G ratio: ↑; UF60: 1–21 d of age: FI: -, BW gain: ↓, F:G ratio: ↑	[46]
Cobb 500, mixed sex	GP	100	-	42	1–42 d of age: FI: -, BW gain: ↓, F:G ratio: -	[47]
Ross 308, male	GP	5, 7.5 or 10	-	28	1–28 d of age: FI: -, BW gain: -, F:G ratio: -	[48]
Cobb 500, unsexed	GS extract	0.25 or 0.5	-	28	0.25: 1–28 d of age: FI: ↑, BW gain: ↑, F:G ratio: ↓; 0.5: 1–28 d of age: FI: ↑, BW gain: ↑, F:G ratio: -	[49]
Cobb 500, mixed sex	GS	10, 20 or 40	GAE: 0.5, 1 or 2 g	42	10: 1–42 d of age: FI: -, BW gain: ↑, F:G ratio: - 20: 1–42 d of age: FI: -, BW gain: ↑, F:G ratio: ↓ 40: 1–42 d of age: FI: -, BW gain: ↓, F:G ratio: ↑	[50]
Ross 308, unsexed	GS extract	0.125, 0.25, 0.5, 1 or 2	-	42	0.125, 0.25, 0.5, 1 and 2: 1–42 d of age: FI: -, BW gain: -, F:G ratio: -	[51]
Cobb 500, male	Grape proanthocyanidins	0.0075, 0.015 or 0.03	-	42	0.0075, 0.015 and 0.03: 1–42 d of age: FI: ↓, BW gain: -, F:G ratio: ↓	[52]
Cobb 500, unsexed	GP	1 g/L drinking water	-	42	1–42 d of age: FI: -, BW gain: -, F:G ratio: -	[53]
Cobb 500, male	GS extract	0.025, 0.25, 2.5 or 5	TEP: 6.6, 66, 660 or 1320 mg	21	0.025, 0.25, 2.5: 1–21 d of age: FI: -, BW gain: -, F:G ratio: -; 5: 1–21 d of age: FI: -, BW gain: ↓, F:G ratio: ↑	[54]
Ross 308, female	GS extract	1	-	42	22–42 d of age: FI: -, BW gain: -, F:G ratio: -	[55]
Cobb 500, male	GS extract (GSE) or GP concentrate (GPC)	7.2 (GSE) or 60 (GPC)	TEP: 5.4 g (GSE) and 4.3 g (GPC)	21	GSE: 1–21 d of age: FI: -, BW gain: ↓, F:G ratio: -; GPC: 1–21 d of age: FI: -, BW gain: -, F:G ratio: ↓	[56]
Hubbard, male	GS extract	0.005, 0.01, 0.02, 0.04 or 0.08	-	15	1–15 d of age: FI: -, BW gain: ↑, F:G ratio: -	[57]
Cobb, male	GP concentrate	15, 30 or 60	TEP + THP: 16.5, 19.2 and 22.1 g in groups 15, 30 and 60	21	21–42 d of age: FI: -, BW gain: -, F:G ratio: -	[58]
Cobb, male	GP	5, 15 or 30	GAE: 4.1 g (GP30)	21	1–21 d of age: FI: -, BW gain: -, F:G ratio: -	[59]

Abbreviations: BW, body weight: FI, feed intake; F:G-ratio, feed:gain-ratio; GAE, gallic acid equivalents; GK, grape skins; GP, grape pomace; GS, grape seeds; TEP, total extractable polyphenols; THP, total hydrolyzable polyphenols.

**Table 2:** Overview of feeding studies reporting effects of grape products on apparent ileal digestibility (AID) or apparent total tract digestibility (ATTD) of protein and/or amino acids in fast-growing broilers.

Broiler Breed and Sex	Grape By-Product	Dose (g/kg Diet)	Main Effects	Ref.
Cobb 500, male	GS or GK	30 (GS) or 110 (GK)	AID of protein: GS: -, GK: ↓	[41]
Cobb 500, male	Fermented or unfermented GK	30 or 60	AID of protein: fermented 30, fermented 60 and unfermented 30: -; unfermented 60: ↓	[46]
Ross 308, male	GP	5, 7.5 or 10	ATTD of crude protein: 5, 7.5 and 10: -	[48]
Cobb, male	GP	50 or 100	AID of protein: 50: -, 100: ↓	[63]
Cobb, male	GS extract	0.025, 0.25, 2.5 or 5	AID of protein: 0.025: ↑, 0.25 and 2.5: -, 5: ↓, AID of arginine, histidine, phenylalanine, glutamic acid, cysteine, proline: 0.025–2.5: -; 5: ↓, AID of other amino acids: 0.025–5: -	[54]
Ross 308, female	GS extract	1	AID of nitrogen: -	[55]
Cobb, male	GP concentrate	15, 30 or 60	15, 30 and 60: AID of protein: -; AID fat: -	[58]
Cobb, male	GP	5, 15 or 30	5, 15 and 30: AID of amino acids: -	[59]

Abbreviations: GK, grape skins; GP, grape pomace; GS, grape seeds.

**Table 3:** Effective dosage ranges for the performance enhancement and performance inhibition of various grape by-products in feed for broilers.

Grape By-Product	Dosage Range for Performance Enhancement (g/kg Diet)	Dosage Range for Performance Inhibition (g/kg Diet)
Grape pomace	5–30	≥60
Grape pomace extract	2	- *
Grape seeds	5–30	≥40
Grape seed extract	0.01–0.5	≥5
Grape skins	- *	≥60

* Not reported.
